# Agreement between chromogenic *in situ* hybridisation (CISH) and FISH in the determination of HER2 status in breast cancer

**DOI:** 10.1038/sj.bjc.6600943

**Published:** 2003-05-13

**Authors:** L Arnould, Y Denoux, G MacGrogan, F Penault-Llorca, M Fiche, I Treilleux, M C Mathieu, A Vincent-Salomon, M O Vilain, J Couturier

**Affiliations:** 1 Department of Pathology, Centre GF Leclerc, 1 rue Pr Marion, 21034 Dijon cedex, France; 2 Department of Pathology, Centre F Baclesse, Caen, France; 3 Institut Bergognie, Bordeaux, France; 4 Centre J Perrin, Clermont-Ferrand, France; 5 Hôpital G et R Laennec, Nantes, France; 6 Centre L Berard, Lyon, France; 7 Institut G Roussy, Villejuif, France; 8 Institut Curie, Paris, France; 9 Centre O Lambret, Lille, France

**Keywords:** breast cancer, HER2, gene amplification, CISH, FISH, immunohistochemistry

## Abstract

Determination of the HER2/neu (HER2) status in breast carcinoma has become necessary for the selection of breast cancer patients for trastuzumab therapy. Amplification of the gene analysed by fluorescence *in situ* hybridisation (FISH) or overexpression of the protein determined by immunohistochemistry (IHC) are the two major methods to establish this status. A strong correlation has been previously demonstrated between these two methods. However, FISH is not always feasible in routine practice and weakly positive IHC tumours (2+) do not always correspond to a gene amplification. Our study was performed in order to evaluate the contribution of chromogenic *in situ* hybridisation (CISH), which enables detection of the gene copies through an immunoperoxidase reaction. CISH was performed in 79 breast carcinomas for which the HER2 status was previously determined by IHC and FISH. The results of IHC, FISH and CISH were compared for each tumour. CISH procedures were successful in 95% of our cases. Whatever the IHC results, we found a very good concordance (96%) between CISH and FISH. Our study confirms that CISH may be an alternative to FISH for the determination of the gene amplification status in 2+ tumours. Our results allow us to think that, in many laboratories, CISH may also be an excellent method to calibrate the IHC procedures or, as a quality control test, to check regularly that the IHC signal is in agreement with the gene status.

Determination of HER2 status has now become of major clinical importance with the advent of anti-HER2 therapy, the recombinant humanised anti-p185^Her-2/neu^ antibody trastuzumab (Herceptin®) (Pegram *et al*, 1998; Cobleigh *et al*, 1999; Slamon *et al*, 2001).

Immunohistochemistry (IHC) is expected to be the best method for the determination of HER2 status, as IHC assesses the level of HER2 overexpression, which is the target of Herceptin® therapy. Moreover, the patient's selection for Herceptin® therapy is mainly based on IHC because previous studies demonstrated a good correlation between IHC results and gene status, as determined by fluorescence *in situ* hybridisation (FISH) (Pegram *et al*, 1998; Jacobs *et al*, 1999,^2000^; Couturier *et al*, 2000; Jimenez *et al*, 2000; Lebeau *et al*, 2001; Lehr *et al*, 2001). However, the HER2-IHC detection was criticised because of a lack of interlaboratory reproducibility and, furthermore, Herceptest®, a standardised IHC method, was shown to be a method with excessive sensitivity when compared to FISH (Persons *et al*, 1997; Bartlett *et al*, 2001; Tubbs *et al*, 2001). Even though HER2 overexpression without gene amplification was reported in 2.9–8.3% of cases (Kallioniemi *et al*, 1992; Persons *et al*, 1997; Couturier *et al*, 2000; Jimenez *et al*, 2000; Pauletti *et al*, 2000), discordant results between IHC and FISH were mainly observed for tumours that were scored 2+ by IHC (Persons *et al*, 1997; Bartlett *et al*, 2001; Tubbs *et al*, 2001). For this reason, and particularly in Europe, a confirmation of HER2 gene amplification by FISH became mandatory for a patient's inclusion in a clinical trial using Herceptin®, when the corresponding tumour is scored 2+ by IHC (Hoang *et al*, 2000; Ridolfi *et al*, 2000; Diaz, 2001; Tubbs *et al*, 2001; Vogel *et al*, 2002).

Some authors found that HER2 status determined by FISH was more reproducible (Press *et al*, 1994; Persons *et al*, 1997; Bartlett *et al*, 2001; Tubbs *et al*, 2001). Thus, these authors thought that FISH had to be proposed as the only method to select patients for Herceptin®. However, FISH is a long and expensive procedure that requires trained personnel and fluorescence microscopy.

Chromogenic *in situ* hybridisation (CISH) is a recently introduced technique in which the DNA probe is detected using an immunoperoxidase reaction (Tanner *et al*, 2000). This method is very close to FISH but does not require the use of fluorescence microscopy. Moreover, FISH signals fade within a few weeks and the FISH results have to be recorded with expensive digital systems. This is not the case for CISH staining. Owing to the similarity with IHC staining, CISH is also easier to interpret for pathologists who are not trained with fluorescence. In one study (Tanner *et al*, 2000), CISH was demonstrated to be well correlated with FISH.

The aims of our study were to: (a) confirm the good correlation between FISH and CISH in a nonhomogeneous series of breast tumours coming from eight different laboratories using different fixation procedures, (b) analyse this correlation according to the expression of HER2 protein analysed by IHC (c) focus on IHC 2+ cases and analyse in this situation if CISH gives the same information as FISH for the treatment of patients.

## MATERIALS AND METHODS

### Tumours

A total of 79 tumours were collected from eight French laboratories. Each laboratory selected cases in which IHC and FISH were previously and successfully performed. In order to analyse the discriminating power of CISH in difficult cases, tumours scored as 2+ by IHC were chosen in priority for this study. Owing to differences in the fixative procedure between the laboratories, 47 tumours were fixed in neutral-buffered formalin, 10 in Holland's bouin, and 22 in alcohol–formalin–acetic acid (AFA).

### IHC

The monoclonal antibody CB11 (Novocastra, Newcastle, England) was used in 25 cases and the polyclonal antibody A485 (Dako, Glostrup, Denmark) in 44 cases. For all slides, immunostaining was scored according to the Herceptest® scoring system, which is also used in clinical trials (Cobleigh *et al*, 1999; Slamon *et al*, 2001). Negativity of normal glands was the prerequisite for interpreting the cases, according to the recommendations of the College of American Pathologists (Fitzgibbons *et al*, 2000).

### FISH

**Figure 1:**
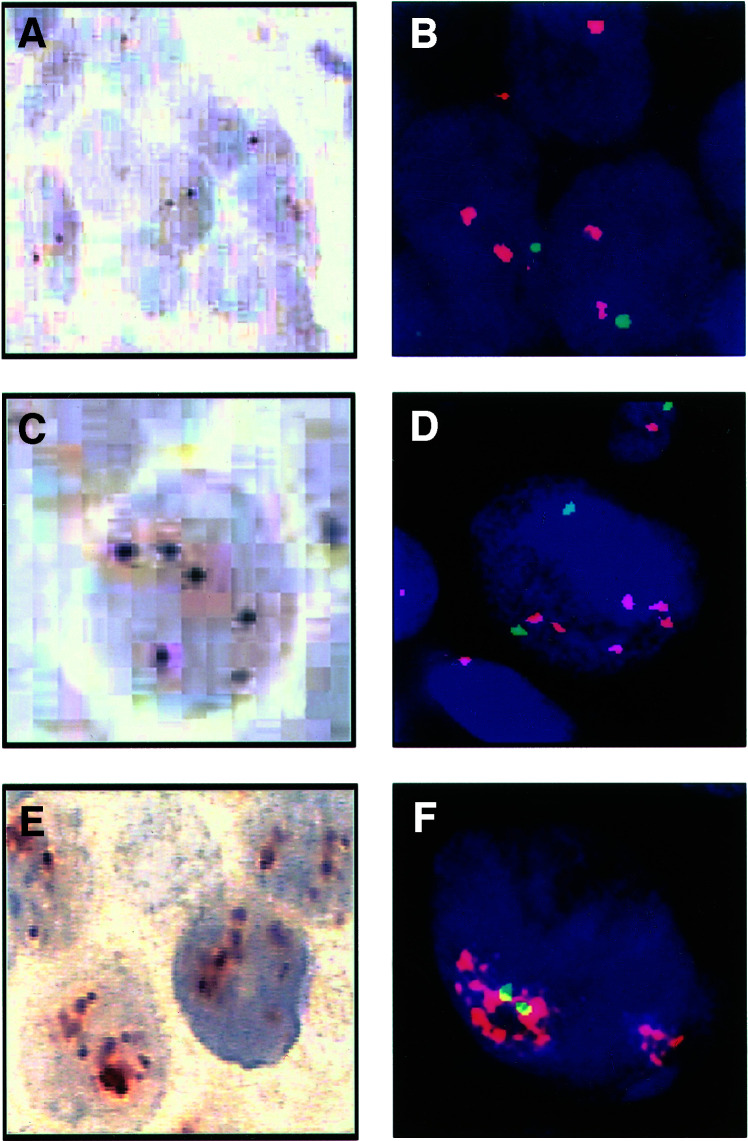
(**A**) CISH, only one or two signals are present in the nucleus of tumour cells (NAC). (**B**) Same case analysed with FISH (NAF). Pink dots correspond to HER2 probe and green dots correspond to centromere 17 probe. (**C**) CISH, six signals are present in the nucleus of tumour cells (LAC). (**D**) Same case analysed with FISH (LAF) with a ratio of HER2 dots/centromere 17 dots=3. (**E**) CISH, large gene copy clusters are present in the nucleus of tumour cells (HAC). (**F**) Same case analysed with FISH (HAF).

FISH was performed in three different referent laboratories. It was performed on frozen tumour sections in 65 cases and on fixed-paraffin-embedded samples in the 14 remaining cases. FISH experiments were performed according to the protocol given by the supplier (PathVysion kit, Vysis, Downers Grove, IL or Ventana HER2 inform, Tucson, AZ). The centromeric probe of chromosome 17 was included in FISH analyses in 62 cases. In these 62 cases, HER2 amplification was determined as a ratio of HER2 and chromosome 17 centromere signal counts. As in the study previously published (Tanner *et al*, 2000), ratios <2 were determined as no amplification with FISH (NAF) (Figure 1B

), those between 2 and 5 as low-level amplification with FISH (LAF) (Figure 1D) and those >5 as high-level amplification with FISH (HAF) (Figure 1F). In the 14 other cases, without centromeric 17 analysis, like for the CISH analysis in the study previously published (Tanner *et al*, 2000), HER2 gene was judged as NAF when 1–5 signals were present per nucleus. When 6–10 signals were present in more than 50% of tumour cell nuclei, the tumours were judged as LAF. Finally, tumours having more than 10 signals in more than 50% of the nuclei were judged as HAF.

### CISH

CISH experiments were performed according to the protocol given by the supplier (Zymed Inc., South San Francisco, CA, USA). The interpretation of the signal was that used by other authors (Tanner *et al*, 2000) and was only performed on invasive tumour patterns. HER2 gene was judged as nonamplified with CISH (NAC) when 1–5 signals were present per nucleus (Figure 1A).When 6–10 signals were present in more than 50% of tumour cell nuclei, the tumours were judged as having a low level of amplification with CISH (LAC) (Figure 1C). Finally, the tumours with more than 10 signals or with large gene copy clusters in more than 50% of the nuclei were judged as having a high level of HER2 gene amplification (HAC) (Figure 1E).

## RESULTS

Evaluation of IHC staining, FISH and CISH signals were performed in a blinded manner.

### IHC

A total of 27 (34%) tumours were defined as 0 or 1+, 29 (37%) tumours were defined as 2+, and the remaining 23 (29%) tumours were defined as 3+ (Table 1

**Table 1:** HER2 gene amplification determined by FISH and CISH, according to the overexpression of HER2 protein determined by IHC

	**IHC**					
	**0 or 1+**	**2+**			**3+**	
	**(*N*=27)**	**(*N*=29)**			**(*N*=23)**	
FISH	27 NAF	14 NAF	11 LAF	4 HAF	1 NAF	22 HAF
						
CISH	25 NAC	align="center"12 NAC	8 LAC	4 HAC	1 NAC	20 HAC
	2 WS	1 LAC	2 HAC			1 NAC
		1 HAC	1 WS			1 WS

NAF=no amplification with FISH; LAF=low level of amplification with FISH; HAF=high level of amplification with FISH; WS=without any signal; NAC=no amplification with CISH; LAC=low level of amplification with CISH; HAC=high level of amplification with CISH.

).

### FISH

As a result of the material chosen for this study, FISH analysis was necessarily successful in all cases. In all, 41 tumours were determined as NAF, 11 tumours as LAF and 26 tumours as HAF (Table 1).

### CISH

CISH was successful in 75 of the 79 tumours (94.9%). The four cases without any signal (WS) corresponded to four of the 22 tumours fixed in AFA. In these tumours, despite the use of a great variation of pretreatment procedures, no signal was present in any tumour cell. In all, 39 tumours were determined as NAC, nine as LAC and 27 as HAC (Table 1).

### Comparison of CISH and FISH results

Only 75 tumours were available for this comparison (Table 1). When we compared all CISH amplifications (LAC+HAC) to all FISH amplifications (LAF+HAF), an agreement was found in 72 out of 75 (96%) tumours (Table 3

**Table 3:** Advantages and disadvantages of FISH and CISH

**FISH**	**CISH**
*Pro*	*Con*
• Possibility of multicolour (HER2 and chromosome 17)	• No multicolour detection
• Is the simplest, without any chromogen detection	• Needs a chromogenic procedure
• Is currently the standard hybridisation procedure for HER2	• Not yet a standard hybridisation procedure for HER2
	
*Con*	*Pro*
• Requires a modern and expensive fluorescence microscope	• An ordinary microscope is effective
• FISH is not routinely used in pathology, and pathologists are not trained to analyse FISH signals	• Pathologists are familiar with IHC signals
• Fluorescence signals can fade within several weeks	• Chromogenic reaction is permanent
• Results have to be recorded with an expensive CCD camera	• Regular slide storage
• Morphology is not always easy to analyse	• Morphology is easier to analyse

). The kappa coefficient (*κ*) measuring agreement between the methods (0: no agreement, 1: agreement) was 0.97 (*P*<10^−9^), and if FISH was chosen as the gold standard, the sensibility of CISH would be close to 97% with a specificity of 95%. In nonproblematic IHC tumours (0, 1+ and 3+), this agreement was found in 46 out of 47 (98%) cases (*κ*=0.95 (*P*<10^−9^), sensibility=95% and specificity=100%). On the other hand, in problematic tumours (2+), this agreement was found in 26 out of 28 (92.8%) cases (*κ*=0.85 (*P*<5 × 10^−6^), sensibility=100% and specificity=85%). When we compared the level of amplification determined with the two methods, an agreement was found in 70 out of 75 (93.3%) with a *κ*=0.88 (*P*<10^−9^).

### Analysis of the cases with polysomy of chromosome 17

Centromeric probes of chromosome 17 were included in FISH analyses in 61 of the 75 cases successfully analysed with CISH. The result of the analysis of this centromere is summarised in Table 2

**Table 2:** Analysis of the cases with or without polysomy of chromosome 17

	**IHC**					
	**0 or 1+**	**2+**			**3+**	
	***N*=24**	***N*=16**			***N*=21**	
Normal chromosome 17 status	*N*=*18*	*N*=*6*			*N*=*20*	
FISH	18 HAF	4 NAF	2 LAF		1 NAF	19 HAF
CISH	18 HAC	4 NAC	2 LAC		1 NAC	18 HAC
						1 NAC
						
Chromosome 17 polysomy						
FISH	*N*=*6*	*N*=*10*			*N*=*1*	
	6 NAF	6 NAF	1 LAF	3 HAF		1 HAF
CISH	6 NAC	5 NAC	1 LAC	3 HAC		1 HAC
		1 LAC				

NAF=no amplification with FISH; LAF=low level of amplification with FISH; HAF=high level of amplification with FISH; WS=without any signal; NAC=no amplification with CISH; LAC=low level of amplification with CISH; HAC=high level of amplification with CISH.

. Using a *χ*^2^ test, we found that polysomy was statically (*P*<0.005) more frequently observed in IHC 2+ tumours (10 out of 16: 62.5%) than in other situations (7 out of 45: 15.55%).

## DISCUSSION

Since the FDA approved Herceptin® for the treatment of metastatic breast cancer (Pegram *et al*, 1998; Cobleigh *et al*, 1999; Roche and Ingle, 1999; Slamon *et al*, 2001), and in order to determine whose patients might benefit from this new therapy, there has been a need to evaluate precisely the HER2 status of breast cancer specimens. The determination of this status will also be important to choose the adjuvant strategy if clinical trials including Herceptin® as adjuvant therapy give a positive result (Hortobagyi and Perez, 2001). Moreover, in the future, HER2 status may also help select patients for tyrosine kinase inhibitor therapy (Moasser *et al*, 2001). Two major methods (IHC and FISH) for the determination of this HER2 status have been developed all around the world but there is no consensus up to now regarding the best methods to determine this status (Thor, 2001). In two recent different clinical trials, poor concordance was found between local and central or reference IHC testing for HER2 (Paik *et al*, 2002; Roche *et al*, 2002). This poor concordance being given, the authors of these studies recommended that the HER2 status of patients included in clinical trials should be done in large-volume reference laboratories. These data also suggested an urgent need to improve the quality control programme in laboratories that use IHC testing (Fitzgibbons *et al*, 2000; Hoang *et al*, 2000; Ridolfi *et al*, 2000; Tubbs *et al*, 2001; Paik *et al*, 2002; Roche *et al*, 2002; Vogel *et al*, 2002). In a previous study, we showed that FISH may be used to obtain successfully a calibration of the in-house IHC technique (Vincent-Salomon *et al*, 2003).

CISH, a hybridisation procedure using a staining of the probe similar to IHC staining, has been previously proposed as an alternative for FISH (Tanner *et al*, 2000). When compared with FISH, CISH has been described as having several advantages (Tanner *et al*, 2000; Zhao *et al*, 2002). It does not require an expensive fluorescence microscope with multi-band-pass filters, CISH staining is permanent and it does not need to be recorded with an expensive CDD camera. Moreover, morphology is easier analysed on CISH slides, particularly for distinguishing invasive cancer cells and *in situ* components. Finally, pathologists are more familiar with the IHC signal than with the FISH signal. The advantages and disadvantages of these two hybridisation techniques are summarised in Table 3.

In order to confirm the results of this study and to precise the place of this technique in problematic IHC cases (2+), we performed a study on a group of 79 breast tumours that contained an abnormal percentage (37%) of 2+. Our study was also different from the study published earlier because the breast tissue came from different purveyor laboratories using different fixatives. CISH procedures were successful in 95% of our cases, which is identical to the results observed with FISH on paraffin sections (Lebeau *et al*, 2001) and very close to the results (98%) published with CISH in an unselected group of tumours (Tanner *et al*, 2000). It may be noticed that AFA seems to be a less effective fixative procedure for CISH, as CISH was successful in only 18 (82%) of the 22 tumours fixed in AFA and successful in all (100%) of the tumours fixed in neutral-buffered formalin or Holland's bouin. We found a very good concordance between the CISH and FISH. In terms of amplification, there was indeed an agreement between CISH and FISH in 96% of the tumours, which is almost the same as the agreement previously published (93.6%) (Tanner *et al*, 2000). The agreement was a little higher (98%) for nonproblematic (0, 1+, 3+) IHC cases than for problematic (2+) IHC tumours (93%). Owing to the small number (3%) of 2+ cases in their study, Tanner *et al* did not notice this small difference. In terms of sensitivity, when we compared the level of amplification estimated by the two methods (no amplification, low level and high level of amplification), we found that the results of CISH analyses were very close to those given by FISH, with an agreement in 93% of the tumours. According to Tanner *et al* (2000), the discrepancy between CISH and FISH may be because of a lower sensitivity of CISH. However, this explains only one discordant case in our study, which was found to be amplified with FISH but not with CISH. Other discrepancies may also be because of differences in the sample materials or the thickness of the slides. The most difficult situations with CISH are when 6–10 spots are present in tumour cells. Double-colour FISH analyses may give more information, particularly the ratio between HER2 signal and the number of chromosome 17, and may separate the high polysomy of chromosome 17 and the very low level of HER2 amplification. We found that polysomy of chromosome 17 is statically more frequent in IHC 2+ tumours, but only one of our discordant cases could probably be linked to this phenomenon. In routine, these situations are very infrequent and it is not proved that this distinction is relevant in terms of response to Herceptin® therapy. Clinical trials, including a large number of IHC 2+ tumours with a low level of amplification, are needed to confirm that the exact level of HER2 gene amplification is important for the patient's selection for specific therapy. Anyway, double-colour staining CISH procedures, including HER2 and chromosome 17 probes, will soon be available. The results of these new procedures would also have to be compared with the double-colour FISH analysis.

Our study confirms that CISH may be an alternative to FISH for the determination of HER2 gene status, particularly in laboratories that are not equipped or trained from fluorescence analyses. In our opinion, CISH is too expensive and too sophisticated to be an alternative to IHC screening of all the breast tumours. However, because of the good correlation between CISH and FISH, even in ambiguous IHC results, we think that it may be used for the determination of gene amplification status in IHC 2+ tumours. Owing to the poor concordance between HER2 status established in local laboratories in comparison to reference laboratories, we also think that, in many laboratories, CISH may be an excellent method to calibrate IHC procedures or, as a quality control test, to check regularly that the IHC signal is in agreement with gene status.
